# Numerical simulation study on the energy dissipation characteristics of energy-dissipating pile-anchor structures

**DOI:** 10.1371/journal.pone.0353460

**Published:** 2026-07-24

**Authors:** Ren Wang, Tong Luo, Xuecheng Liu, Tao Zhou, Xuanming Ding, Bo Zhang

**Affiliations:** 1 School of Civil Engineering, Chongqing University, Chongqing, China; 2 Beijing Urban Construction Design and Development Group Co., Limited, Beijing, China; 3 China Railway First Survey and Design Institute Group Ltd., Xian, China; 4 Hunan Engineering Research Center for Disaster Evolution and Reinforcement of Deteriorating Structures, Hunan Institute of Engineering, Xiangtan, China; 5 China Railway Yangtze River Transportation Design Group Co., Ltd, Chongqing, China; Hohai University, CHINA

## Abstract

This study investigates the energy dissipation mechanisms and damage evolution of energy-dissipating and ductile pile-anchor structures under seismic loading through numerical simulation. A three-dimensional finite element model was developed, incorporating the Concrete Damaged Plasticity model for simulating concrete behavior, a microscopic model for Fiber-Reinforced Concrete, specifically Engineered Cementitious Composite, and a static-dynamic boundary conversion method to enhance analysis accuracy. A series of parametric studies were conducted to analyze the effects of pile stiffness, pile-soil interface roughness, and the use of Engineered Cementitious Composite ductile members on the structural response. Key findings indicate that: (1) The Concrete Damaged Plasticity model combined with the 3D microscopic model effectively simulates the dynamic damage characteristics of concrete and Engineered Cementitious Composite. (2) Increasing pile stiffness reduces pile damage but can increase soil plastic deformation beyond an optimal point, highlighting the need for balanced stiffness and deformation capacity. (3) Increasing the pile-soil friction coefficient alters the failure mode and energy dissipation pattern of the pile due to changes in eccentric axial force and additional bending moment. (4) Engineered Cementitious Composite members significantly reduce structural damage and enhance energy dissipation capacity, particularly under strong seismic motions, by maintaining integrity and fully activating damping devices.

## 1. Introduction

Earthquakes, as natural disasters characterized by sudden occurrence and high destructive power, pose a serious threat to the safety of engineering infrastructure in human society [1–4]. Particularly in mountainous and hilly regions, earthquakes are highly prone to inducing secondary geological hazards such as landslides and collapses [5,6]. Seismic landslides, one of the primary secondary geological hazards triggered by earthquakes, exhibit characteristics such as extensive impact range, severe damage levels, and a tendency to form disaster chains [7,8]. Therefore, to reduce casualties and infrastructure losses caused by seismic landslides, enhance the seismic performance of slope support structures under earthquake conditions, and ensure their safety and stability, research on seismic landslide mitigation has become a crucial topic in the fields of geotechnical engineering and earthquake engineering [9]. As a widely used support structure in the prevention and control of seismic landslides, the seismic performance of anchor-reinforced anti-slide piles has attracted significant attention from numerous researchers [10–14].

Currently, Scholars have conducted thorough investigations into the response patterns and failure mechanisms of anchored anti-slide piles under seismic action through approaches such as model testing and theoretical analysis [15]. Shi et al. [16] proposed the use of the force method to calculate the axial force of anchor cables, aiming to determine the proportion of landslide thrust borne by the anchor cables and the anti-slide piles. Model testing, as a crucial means of studying structural seismic responses at the current stage, has been widely employed by numerous scholars in researching the response characteristics of anchored anti-slide piles under seismic action. Wang et al. [17] investigated the influence of different anchor layouts on the deformation characteristics of anchored anti-slide piles through physical model tests. Lian et al. [18] explored the evolution of dynamic characteristics of anchored anti-slide pile systems under seismic conditions from multiple perspectives using large-scale shaking table tests. Wu et al. [19] analyzed the seismic dynamic response of bedding rock slopes reinforced with pile-anchor structures through shaking table tests. However, experimental methods have obvious limitations: on one hand, constrained by time, cost, and experimental conditions, it is difficult to conduct systematic studies covering multiple parameters and working conditions. Furthermore, test models often cannot fully simulate the initial in-situ stress state, infinite domain radiation damping effects, and complex pile-soil interactions in actual engineering, all of which may affect the universality and accuracy of research conclusions.

In contrast, numerical simulation technology, with its advantages of low cost, controllable parameters, repeatable processes, and refined result extraction, has become an indispensable supplement or even a leading means for studying the seismic performance of complex geotechnical-structural systems [20]. Particularly, three-dimensional nonlinear finite element methods can realistically simulate material nonlinearity (e.g., concrete cracking, soil plasticity), contact nonlinearity (e.g., pile-soil interface slip, separation), and boundary conditions (e.g., artificial boundaries simulating infinite domains), providing a powerful tool for in-depth analysis of the energy flow paths, damage accumulation mechanisms, and failure modes of energy-dissipating pile-anchor structures throughout the seismic process. Among these, selecting appropriate material constitutive models is key to ensuring simulation accuracy. Wang et al. [21] used the finite element method to evaluate the anti-sliding effectiveness of prestressed anchored anti-slide piles under seismic action. Li et al. [22] conducted a parameter optimization design for prestressed pile-anchor structures based on the finite difference method, and found that the pile position significantly influences the stability of the reinforced slope. In recent years, various types of high-performance energy dissipation devices have been extensively studied and developed for structural seismic control [23–25]. Despite the extensive application of energy dissipation technologies in bridges and high-rise buildings, their use in slope support systems, particularly pile anchor structures, remains limited. From a research perspective, few studies have examined the dynamic response and energy dissipation mechanisms of such systems under seismic loading. From a development perspective, the absence of standardized design guidelines and experimentally validated numerical models hinders their practical implementation in slope engineering. The accurate simulation of frictional sliding behavior at the pile-soil interface and its influence mechanism on the overall mechanical response remain unclear. Moreover, systematic research on the effects of different design parameters, such as pile stiffness, interface roughness, and material ductility, on the energy dissipation characteristics and damage development of energy-dissipating pile-anchor structures has not yet been sufficiently conducted.

To address these issues, this study adopts numerical simulation methods to conduct an in-depth and systematic investigation into the energy dissipation characteristics and damage evolution patterns of energy-dissipating and ductile pile-anchor structures under seismic action. Through a series of numerical simulations and comparative analyses targeting the aforementioned parameters, the energy dissipation mechanisms and damage evolution laws of energy-dissipating pile-anchor structures are revealed. This research systematically examines the influence of key design parameters, such as pile stiffness, interface roughness, and material ductility, on the energy dissipation properties and deformation-failure modes of the new pile-anchor structures, thereby providing a theoretical basis and data support for the seismic optimization design and engineering application of this novel seismic support structure. The findings will not only contribute to a deeper understanding of the seismic behavior of complex pile-soil-structure systems but also hold significant theoretical importance and engineering value for advancing the development of high-energy-dissipation, high-ductility slope support technologies.

## 2. Finite element numerical analysis

The study was conducted in three phases. First, the damper device was optimized based on preliminary computational fluid dynamics analysis. Second, a three-dimensional finite element model was developed and validated against the results of 1:10 scale shaking table tests reported by Liu et al., which included comparisons of acceleration, bending moment, and displacement. Third, a single-variable parametric analysis was performed to systematically investigate the effects of pile stiffness, pile–soil friction coefficient, and Engineered Cementitious Composite (ECC) material on structural energy dissipation (EPDDEN, EDMDDEN) and damage distribution (DAMAGEC, DAMAGET).

### 2.1. Concrete damaged plasticity model

Concrete, a typical quasi-brittle material, exhibits significant differences in tensile and compressive behavior. Under tensile cracking, crack closure considerably affects the mechanical response. Additionally, under cyclic loading, concrete experiences damage due to repeated loading, leading to significant degradation of strength and stiffness. Given this complex mechanical behavior, the Concrete Damaged Plasticity (CDP) model was selected to simulate the stress-strain relationship of the anti-slide piles.

The CDP constitutive model was initially proposed by Lubliner [26] in the late 1980s. It was subsequently modified and improved by Lee and Fenves [27] among others, and has been adopted by the ABAQUS software for both explicit and implicit analyses. Based on the concepts of damage mechanics, the model introduces damage variables to characterize material degradation and incorporates stiffness recovery effects under cyclic loading. This enables an accurate description of the initiation, evolution, and macroscopic failure process of material damage. In addition to damage behavior, the CDP model integrates plasticity theory to simulate the plastic response of materials, thereby providing a reliable framework for modeling the mechanical behavior of concrete under dynamic loading.

#### 2.1.1. Damage behavior.

**Fig 1:**
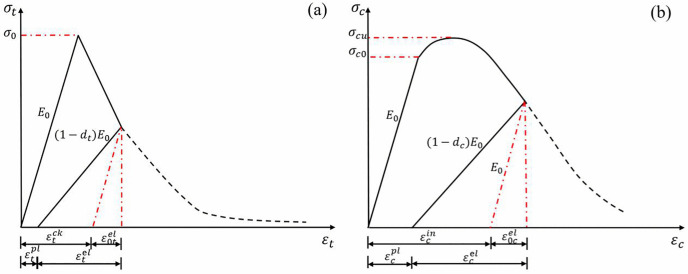
Uniaxial tensile and compressive stress-strain relations of concrete (a) Uniaxial tensile stress-strain curve of concrete; (b) Uniaxial compressive stress-strain curve of concrete.

The CDP model assumes material failure modes are primarily tensile cracking and compressive crushing. The failure surface shape is controlled by equivalent plastic strain, which is closely related to damage characteristics under uniaxial loading. Fig 1 illustrates the assumed uniaxial damage mechanism of concrete in the CDP model.

Fig 1 shows the deformation characteristics of concrete under uniaxial loading. The CDP model simplifies the uniaxial tensile stress-strain relationship into an elastic ascending branch and a descending branch, while the uniaxial compressive stress-strain relationship is simplified into an elastic branch, a hardening branch, and a descending branch. This simplification aligns with the main deformation characteristics of concrete.

Furthermore, the CDP model introduces damage degradation variables (*d*_*c*_ and *d*_*t*_) into the uniaxial stress-strain relationship to reflect the effect of damage on material stiffness. The stress-strain relations are expressed as:


$$\begin{array}{c} \sigma_{t}=(1-d_{t})E_{\text{0}}(\varepsilon_{t}-\varepsilon_{t}^{pl}) \end{array}$$ (1)

$$\begin{array}{c} \sigma_{c}=(1-d_{c})E_{\text{0}}(\varepsilon_{c}-\varepsilon_{c}^{pl}) \end{array}$$ (2)

Where $\sigma_{t}$ and $\sigma_{c}$ represent tensile and compressive stress; $\varepsilon_{t}$ and $\varepsilon_{c}$ represent tensile and compressive strain; $\varepsilon_{t}^{pl}$ and $\varepsilon_{c}^{pl}$ are tensile and compressive equivalent plastic strains; *d*_*c*_ and *d*_*t*_ are tensile and compressive damage degradation factors; $E_{\text{0}}$ is the initial elastic modulus. $\varepsilon_{t}^{pl}$ and $\varepsilon_{c}^{pl}$ are calculated as:


$$\begin{array}{c} \varepsilon_{t}^{pl}=\varepsilon_{t}^{ck}-\frac{d_{t}}{1-d_{t}}\frac{\sigma_{t}}{E_{\text{0}}} \end{array}$$ (3)

$$\begin{array}{c} \varepsilon_{c}^{pl}=\varepsilon_{c}^{in}-\frac{d_{c}}{1-d_{c}}\frac{\sigma_{c}}{E_{\text{0}}} \end{array}$$ (4)

Where $\varepsilon_{t}^{ck}$ and $\varepsilon_{t}^{in}$ respectively represent the cracking strain and inelastic strain of concrete:


$$\begin{array}{c} \varepsilon_{t}^{ck}=\varepsilon_{t}-\frac{\sigma_{t}}{E_{\text{0}}} \end{array}$$ (5)

$$\begin{array}{c} \varepsilon_{c}^{in}=\varepsilon_{c}-\frac{\sigma_{c}}{E_{\text{0}}} \end{array}$$ (6)

Using the CDP model requires defining both the uniaxial stress-cracking strain (or inelastic strain) relationship and the corresponding relationship between cracking strain (or inelastic strain) and the damage factor. These relationships can be calculated from the uniaxial stress-strain curve, as detailed later.

#### 2.1.2. Hysteretic behavior.

Tests show that concrete exhibits stiffness recovery under cyclic loading because cracks close when the material transitions from tension to compression, partially restoring the elastic modulus. The CDP model uses the damage variable *d* to describe material stiffness degradation. The value of *d* depends on the tensile and compressive damage factors and the stress state:


$$\begin{array}{c} E=(1-d)E_{\text{0}} \end{array}$$ (7)

$$\begin{array}{c} \left(1-d)=(1-s_{t}d_{c})(1-s_{c}d_{t}\right) \end{array}$$ (8)

Where *d*_*c*_ and *d*_*t*_ are tensile and compressive damage degradation factors, reflecting the unloading response under tensile and compressive states, related to cracking strain or inelastic strain; *s*_*t*_ and *s*_*c*_ are stress state functions considering stiffness recovery effects in different stress directions, calculated as:


$$\begin{array}{c} s_{t}=1-\omega_{t}r^{*}\left(\sigma_{11}\right)\text{0}\leq \omega_{t}\leq 1 \end{array}$$ (9)

$$\begin{array}{c} s_{c}=1-\omega_{c}\left(1-r^{*}\left(\sigma_{11}\right)\right)\text{0}\leq \omega_{c}\leq 1 \end{array}$$ (10)

**Fig 2:**
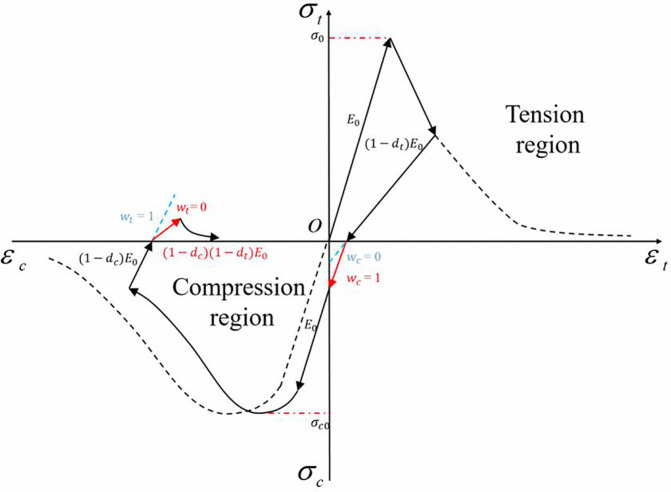
Stress-strain curve under uniaxial cyclic loading (CDP model).

Where $r^{*}\left(\sigma_{11}\right)$ is a function related to the principal stress state, equaling 1 if $\sigma_{11}>0$ and 0 if $\sigma_{11}<0$; *w*_*t*_ and *w*_*c*_ are tensile and compressive stiffness recovery factors, determining the degree of stiffness recovery upon stress state change. Fig 2 depicts the hysteretic behavior of concrete under uniaxial cyclic loading in the CDP model. Since compressive cracks persist during tension, stiffness does not recover, thus $w_{t}=0$. Tensile cracks close under compression, and the CDP model assumes the elastic modulus fully recovers to its initial value, hence $w_{c}=1$.

#### 2.1.3. Yield function and flow rule.

The CDP model uses a yield criterion initially proposed by Lubliner et al. [26] and later modified by Lee and Fenves [27]. This yield function uses equivalent plastic strains $\varepsilon_{t}^{pl}$ and $\varepsilon_{c}^{pl}$ as hardening variables controlling yield surface evolution. The yield function expressed in terms of effective stress is:


$$\begin{array}{c} f=\frac{\text{1}}{1-\alpha }(\overset{―}{q}-3\alpha \overset{―}{p}+\beta (\varepsilon^{pl})\langle {\frac{\wedge }{\sigma }}_{max}\rangle -\gamma \langle -{\frac{\wedge }{\sigma }}_{max}\rangle )-\overset{―}{\sigma_{c}}\left(\varepsilon_{c}^{pl}\right)=0 \end{array}$$ (11)

$$\begin{array}{c} \alpha =\frac{\sigma_{b0}-\sigma_{c0}}{2\sigma_{b0}-\sigma_{c0}} \end{array}$$ (12)

$$\begin{array}{c} \beta \left(\varepsilon^{pl}\right)=\frac{{\overset{―}{\sigma }}_{c}\text{(}\varepsilon_{c}^{pl}\text{)}}{{\overset{―}{\sigma }}_{c}\text{(}\varepsilon_{t}^{pl}\text{)}}(1-\alpha )-(1+\alpha )\text{=0} \end{array}$$ (13)

$$\begin{array}{c} \gamma =\frac{3\text{(1-}K_{c}\text{)}}{2K_{c}-1} \end{array}$$ (14)

Where ${\hat{\bar{\sigma }}}_{\text{max}}$ is the maximum principal stress; $\sigma_{b0}$ and $\sigma_{c0}$ represent the biaxial and uniaxial initial compressive yield stresses, typically taken as 1.16; *K*_*c*_ is the invariant stress ratio influencing the yield surface shape, typically defaulting to 2/3.

The CDP model adopts a non-associated flow rule. The plastic potential function uses the Drucker-Prager hyperbolic function:


$$\begin{array}{c} d\varepsilon_{ij}^{p}=d\lambda \frac{\partial g}{\partial \sigma_{ij}} \end{array}$$ (15)

$$\begin{array}{c} g=\sqrt{(e\sigma_{t0}tan\psi {)}^{\text{2}}+{\overset{―}{q}}^{\text{2}}}-\overset{―}{p}tan\psi \end{array}$$ (16)

Where ψ is the dilation angle; *e* is the eccentricity, representing the rate at which the plastic potential approaches the asymptote.

To improve computational performance, the CDP model introduces a viscosity parameter *v* to enhance convergence in the softening stage, typically set to 1E-5.

#### 2.1.4. Calculation of model parameters.

As mentioned, key CDP model parameters include elastic parameters (elastic modulus *E* and Poisson’s ratio *v),* plastic parameters (dilation angle ψ*,* eccentricity *e,* stress ratio *K*_*c*_*,* uniaxial/biaxial initial yield stress ratio $\frac{\sigma_{b0}}{\sigma_{c0}}$, viscosity parameter *v,* etc*.*)*,* hardening parameters ($\sigma_{c}-\varepsilon_{c}^{in}$ and $\sigma_{t}-\varepsilon_{t}^{sk}$ relationships)*,* and damage parameters ($\varepsilon_{t}^{ck}-d_{t}$ and $\varepsilon_{c}^{in}-d_{c}$ relationships*).* Note that some parameters, like the dilation angle and eccentricity, cannot be directly determined from material tests. Their selection is crucial for defining the yield surface shape and flow rule. This study references existing research to determine these values, as listed in Table 1.

**Table 1:** Parameter values of CDP model.

Plastic Parameters					Stiffness Recovery Factors	
ψ	e	$K_{c}$	$\sigma_{b0}/\;\sigma_{c0}$	v	$w_{t}$	$w_{c}$
38°	0.1	0.667	1.16	1E-5	0	1

Other parameters are determined from basic material tests. The concrete elastic modulus E is calculated according to the CEB-FIP [28] recommended formula:


$$\begin{array}{c} E=(0.8+0.2\frac{f_{cm}}{88})E_{ci} \end{array}$$ (17)

$$\begin{array}{c} E_{ci}=21.5\times 10^{\text{3}}(\frac{f_{cm}}{10}{)}^{\frac{\text{1}}{\text{3}}} \end{array}$$ (18)

$$\begin{array}{c} f_{cm}=f_{ck}+8 \end{array}$$ (19)

Where *E* is the concrete elastic modulus; *E*_*ci*_ is the concrete secant modulus; *f*_*cm*_ is the mean compressive strength; *f*_*ck*_ is the characteristic cylinder compressive strength. The Poisson’s ratio *v* for concrete is taken as 0.2 according to the code [29].

In the CDP model, hardening and damage parameters depend on the selection of uniaxial tensile and compressive stress-strain curves. Referencing existing research [30], this paper selects a two-segment (ascending and descending) uniaxial compressive stress-strain model:


$$\begin{array}{c} y=\frac{N_{x}-x^{\text{2}}}{1+(N-2)x}\;(x<1) \end{array}$$ (20)

$$\begin{array}{c} y=\frac{x}{\alpha_{c}(x-1{)}^{\text{2}}+x}\;(x\geq 1) \end{array}$$ (21)

$$\begin{array}{c} x=\frac{\varepsilon }{\varepsilon_{c}}\;y=\frac{\sigma }{f_{c}} \end{array}$$ (22)

Where $\varepsilon_{c}$ represents the compressive strain corresponding to the peak compressive strength; *f*_*c*_ is the axial compressive strength. *N* and $\alpha_{c}$ are parameters calibrated from the complete compressive stress-strain curve test. According to research by Guo et al. [31], their values are shown in Table 2.

**Table 2:** Compressive stress-strain curve parameter values.

Concrete strength grade	N	$\alpha_{c}$	$\frac{{𝐄}_{c}}{10}-3$
C20	2.2	0.4	1.4
C30	1.7	0.8	1.6
C40	1.7	2.0	1.8

The uniaxial tensile stress-strain curve is also divided into ascending and descending segments [32]:


$$\begin{array}{c} y=\frac{Ax-x^{\text{2}}}{1+(A-2)x}\;(x<1) \end{array}$$ (23)

$$\begin{array}{c} y=\frac{x}{\alpha_{t}(x-1{)}^{1.7}+x}\;(x\geq 1) \end{array}$$ (24)

$$\begin{array}{c} x=\frac{\varepsilon }{\varepsilon_{t}}\;y=\frac{\sigma }{f_{t}} \end{array}$$ (25)

$$\begin{array}{c} \alpha_{t}=-0.22+0.175f_{cu}^{\frac{\text{2}}{\text{3}}} \end{array}$$ (26)

$$\begin{array}{c} \varepsilon_{t}=3.14\times 10^{-6}f_{cu}^{0.36} \end{array}$$ (27)

Where *A*is taken as 1.306; $\varepsilon_{t}$ represents the tensile strain corresponding to the peak tensile strength; $f_{t}$ is the axial tensile strength; $f_{cu}$ represents the concrete cube compressive strength. After determining the concrete uniaxial stress-strain curve, the $\sigma_{c}-\varepsilon_{c}^{in}$ and $\sigma_{t}-\varepsilon_{t}^{ck}$ relationships can be calculated as:


$$\begin{array}{c} \varepsilon_{in}=\varepsilon_{c}-\frac{\sigma_{c}}{E} \end{array}$$ (28)

$$\begin{array}{c} \varepsilon_{ck}=\varepsilon_{t}-\frac{\sigma_{t}}{E} \end{array}$$ (29)

The damage parameters ($\varepsilon_{t}^{ck}-d_{t}$ and $\varepsilon_{t}^{in}-d_{c}$ relationships) are calculated using the method proposed by Sidoroff [33] based on the energy equivalence principle, which effectively improves computational convergence:


$$\begin{array}{c} d_{c}=1-\sqrt{\frac{\sigma_{c}}{E\varepsilon_{c}}} \end{array}$$ (30)

$$\begin{array}{c} d_{t}=1-\sqrt{\frac{\sigma_{t}}{E\varepsilon_{t}}} \end{array}$$ (31)

Using the above method, the input parameters for the CDP model were obtained. Taking concrete with *E* = 35GPa as an example, the specific values are shown in Tables 3 and 4.

**Table 3:** Relationship of compressive stress $\sigma_{c}$ inelastic strain $\varepsilon_{in}$ compressive damage factor *d*_*c*_.

$\sigma_{c}$	0.00	14.81	21.18	18.44	14.78	12.00	9.98	7.40	3.18
e	0.0000	0.0000	7.00 E-4	1.70 E-3	2.60 E-3	3.50 E-3	4.50 E-3	6.30 E-3	1.48 E-2
$d_{c}$	0.0000	0.0000	0.2936	0.4778	0.6009	0.6811	0.7358	0.8043	0.9149

**Table 4:** Relationship of tensile stress $\sigma_{t}$ cracking strain $\varepsilon_{ck}$ tensile damage factor *d*_*t.*_.

$\sigma_{t}$	0.00	2.43	2.11	1.58	1.04	0.78	0.36	0.15	0.06
$\varepsilon_{ck}$	0.0000	0.0000	5.6E-5	1.3E-4	2.5E-4	3.6E-4	9.8E-4	3.4E-3	1.3E-2
$d_{t}$	0.0000	0.0000	0.2518	0.4511	0.6461	0.7363	0.8886	0.9617	0.9877

### 2.2. Numerical model setup

**Fig 3:**
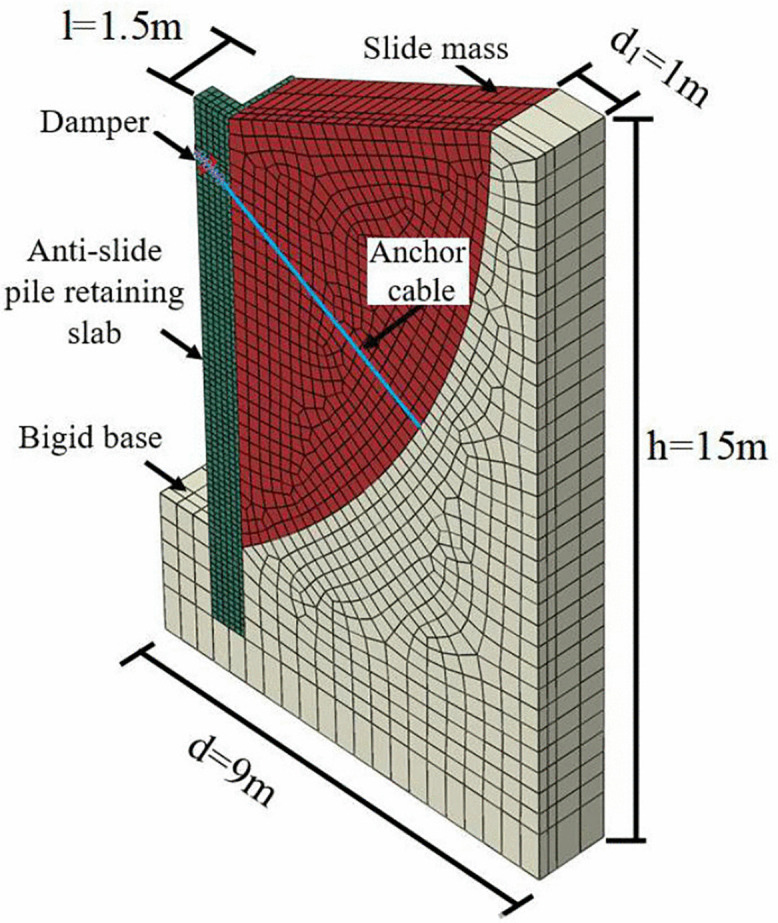
Magnification diagram of the main model area.

This paper analyzes the seismic performance of energy-dissipating pile-anchor structures by establishing a series of 3D finite element models. The computational model is divided into three main regions: the primary model calculation area, an extended calculation area, and an infinite element area. As shown in Fig 3, the primary model area consists of the anti-slide pile, slide mass, bedrock, anchor cable, and damper. The extended calculation area is the underlying bedrock.

The CDP model simulates the dynamic damage characteristics of the anti-slide pile. The soil’s plastic characteristics are modeled using the Mohr-Coulomb constitutive model. Since the bedrock and anchor cables remain elastic in this study, they are simulated using a linear elastic model. The damper is simulated using a Spring-A (axial) element in Abaqus, with its behavior defined by the aforementioned power-law relationship. The anti-slide pile and bedrock are connected with a “tie” constraint. The interface between the anti-slide pile and the slide mass uses a Coulomb friction model, with hard contact in the normal direction and a penalty function in the tangential direction. The friction coefficient is determined by *μ*.

The element types for the anti-slide pile, bedrock, and slide mass are C3D8R. The anchor cable uses T3D2 elements to simulate its tension-only characteristics. Additionally, CIN3D8 infinite elements are established around the model as absorbing boundaries to simulate the radiation damping effect of the infinite foundation [34]. To improve computational efficiency, model symmetry is utilized, modeling only $\frac{\text{1}}{\text{2}}$ of the region. The primary model area is meshed finer for accuracy, and mesh independence verification ensures reliability. The final maximum element size is about 2% of the wavelength, well below the $\frac{\text{1}}{10}$ wave length limit, effectively simulating wave propagation [35].

The dimensions of the pile-anchor structure in the numerical simulation model were derived from the actual dimensions of the 1:10 scale shaking table test model reported in Liu et al. [12]. The anti-slide pile has a cross-section of 0.8 m × 0.8 m, a total length of 11 m, and a fixed-end embedment depth of 2 m. The specific dimensions of the primary model area are back-calculated from the shaking table test model dimensions, but the rock-soil contact surface in the numerical analysis uses an ideal logarithmic spiral slip surface. Modeling and analysis are conducted according to the dimensions in Fig 3, where the Y-direction is the gravity direction and the X-direction is the unidirectional horizontal seismic loading direction. To eliminate the influence of artificial truncated boundaries, the extended area length is ten times that of the primary model area, and the depth is twice that of the primary model area.

#### 2.2.1. Configuration and constitutive model of the viscous damper.

**Fig 4:**
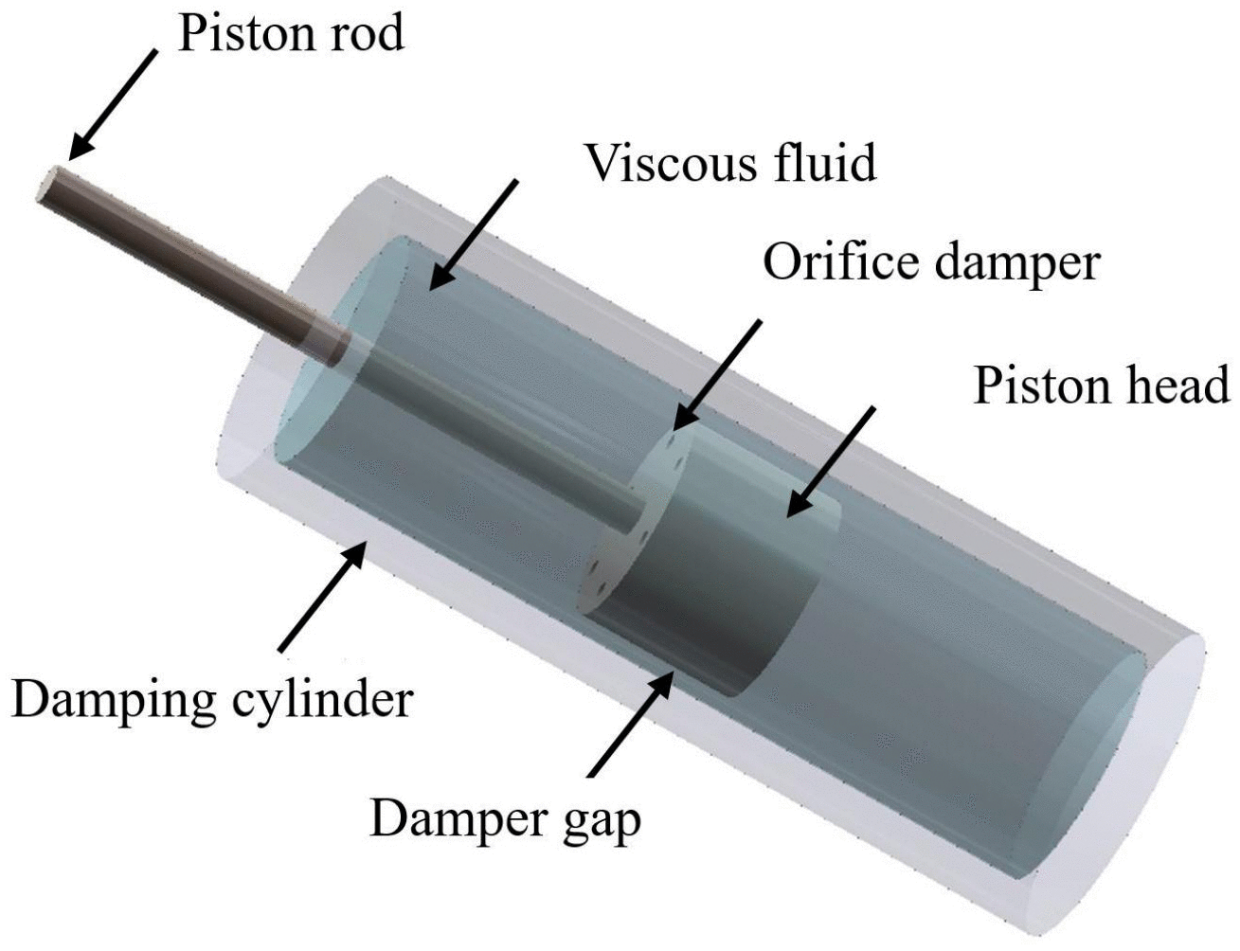
Schematic diagram of the overall structure of a viscous damper.

The energy-dissipating pile-anchor structure in this study incorporates a single-rod gap-type viscous fluid damper, as depicted in Fig 4. The primary components of the damper include the cylinder, a piston head with a specially designed orifice, a highly viscous fluid (typically silicone oil), and the connecting rod. The energy dissipation mechanism is activated by the relative motion between the pile head and the anchor cable during an earthquake. This motion drives the piston to shear the viscous fluid, forcing it to flow through the annular gap between the piston and the cylinder wall or through specifically designed orifices. This process converts mechanical energy into heat, thereby dissipating seismic energy and reducing the dynamic force on the anchor cable.

To accurately simulate its mechanical behavior, the damper is represented in the finite element model using a Spring-A (axial) element in Abaqus. The constitutive relationship of this element is defined by the widely accepted fractional power-law model for fluid viscous dampers [36]. The relationship between the output damping force *F* and the relative velocity across the damper is given by:


$$\begin{array}{c} F=C\cdot sgn\left(\dot{u}\right)\cdot {\left|\dot{u}\right|}^{\alpha } \end{array}$$ (32)

Where *C* is the damping coefficient, which is a function of the fluid viscosity and the geometry of the fluid path, and α is the velocity exponent. The velocity exponent characterizes the damper’s linearity. α=1 represents a linear damper, while 0<α<1 represents a nonlinear damper, which is more common for high-performance seismic applications. The specific values for the damping coefficient C and the velocity exponent used in our simulations (e.g., C=600kN/(m/s) in the parametric study) were calibrated based on detailed computational fluid dynamics (CFD) analyses and experimental data from Liu et al. [12] to ensure they accurately represent a stable and effective energy-dissipating device under strong seismic motions.

### 2.3. Static-dynamic boundary conversion

The static-dynamic coupling method used in numerical calculations directly affects the rationality of the final results. In geotechnical dynamic calculations, the initial stress caused by soil self-weight must be considered. Initial stress calculation typically requires static boundary conditions, while dynamic boundaries like infinite elements used in dynamic calculations are generally not suitable for static analysis. This is because static infinite elements under body forces simulate the constraining support of the infinite domain on the finite domain by introducing boundary nodal forces. However, research shows these nodal forces are not fully equivalent to static boundaries. Applying infinite elements to static calculations can cause deviations in the initial stress state, subsequently affecting the nonlinear response of the model [37]. This creates an inconsistency in boundary conditions before and after the calculation.

**Fig 5:**
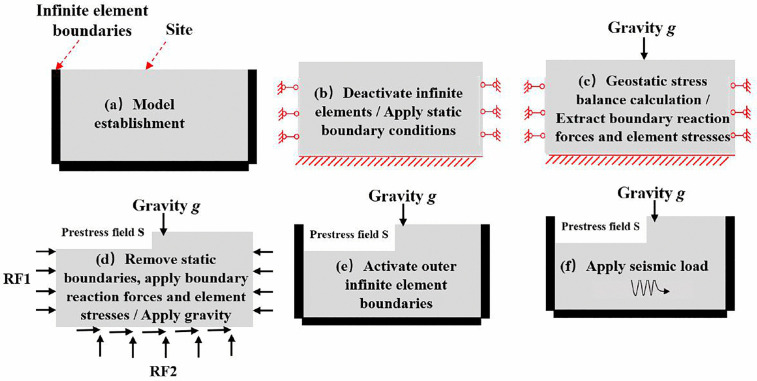
Schematic diagram of dynamic boundary conversion process.

Significant plastic deformation occurs in soil under seismic action, preventing the use of superposition principles to calculate structural responses separately under static and dynamic boundaries and then combine them. Therefore, this study references the research of Wang et al. [37], adopting a static-dynamic boundary conversion method for coupled static-dynamic calculation, as shown in Fig 5.

Due to symmetry, the vertical boundaries perpendicular to the *Z*-axis do not move in the *Z*-direction in both static and dynamic calculations; thus, roller boundaries constraining *Z*-displacement are always applied [38]. The vertical boundaries perpendicular to the *X*-axis use roller boundaries constraining *X*-displacement in static calculation, while the bottom uses a fixed boundary. In dynamic calculation, both are converted to infinite element dynamic boundaries. Note that when using infinite elements as artificial boundaries, displacement-based input cannot be used. If displacement boundary conditions are applied at the bottom, the bottom node displacements become unaffected by reflected waves, causing surface reflections to re-reflect at the bottom, rendering the infinite elements ineffective. Therefore, this study converts the seismic acceleration time history into a stress time history for application [39]:


$$\begin{array}{c} \sigma_{n}=-2\left(\rho C_{n}\right)v_{n} \end{array}$$ (33)

$$\begin{array}{c} \sigma_{s}=-2\left(\rho C_{s}\right)v_{s} \end{array}$$ (34)

Where $\sigma_{n}$ and $\sigma_{s}$ represent the input normal and shear stresses at the bottom; ρ is the density of the propagation medium; $C_{n}$ and $C_{s}$ represent the propagation velocities of compression and shear waves in the medium; $v_{n}$ and $v_{s}$ represent the normal and tangential velocity components. To prevent excessive rigid body displacement, baseline correction of the acceleration time history is necessary before conversion.

### 2.4. Case design

The study reveals that the incorporation of energy-dissipating anchor cables and ductile members significantly influences the mechanical and deformation behavior of pile-anchor structures [12]. To further investigate their effect on dynamic response, a series of numerical analyses studied the seismic response characteristics of energy-dissipating pile-anchor structures with different damper coefficients and ductile pile-anchor structures. Additionally, considering that pile-soil interaction is key to determining the final deformation failure mode, this study systematically analyzes the seismic response characteristics of energy-dissipating anchor cable anti-slide piles with different pile-soil relative stiffness and pile-soil contact roughness to explore their impact on energy dissipation characteristics, pile damage development, and anchor cable axial force response. Pile-soil relative stiffness is adjusted by changing the pile’s elastic modulus, while pile-soil contact roughness is varied by setting the tangential contact friction coefficient, calculated based on the structure-soil friction angle recommended by the “Technical Code for Building Slope Engineering” GB 50330−2013. Specific case designs are shown in Table 5. It should be noted that the damper damping coefficient of 600 kN/(m/s) listed in Table 5 corresponds to the coefficient *C* in the power-law constitutive model described in “Configuration and Constitutive Model of the Viscous Damper”, and it is applied to the Spring-A element representing the damper in the numerical model.

**Table 5:** Numerical example of parametric analysis of seismic response characteristics of energy-dissipating pile-anchor structures.

No.	Pile Material	Pile Elastic Modulus	pile-soil interface friction coefficient	damping coefficient of energy-dissipating anchor cable (kN.s/m))	peak ground acceleratio (PGA)
R-35-30-6-05	Ordinary Concrete	35GPa	0.148	600	0.5g
R-35-30-6-07	Ordinary Concrete	35GPa	0.148	600	0.7g
R-35-30-6-09	Ordinary Concrete	35GPa	0.148	600	0.9g
R-25-30-6-09	Ordinary Concrete	25GPa	0.148	600	0.5g
R-45-30-6-09	Ordinary Concrete	45GPa	0.148	600	0.5g
R-35-40-6-09	Ordinary Concrete	35GPa	0.198	600	0.5g
R-35-50-6-09	Ordinary Concrete	35GPa	0.249	600	0.5g
R-35-60-6-09	Ordinary Concrete	35GPa	0.302	600	0.5g
E-35-30-6-05	ECC Material	35GPa	0.148	600	0.5g
E-35-30-6-07	ECC Material	35GPa	0.148	600	0.7g

### 2.5. Model verification and material parameters

Before parametric analysis, the rationality of the finite element model for the energy-dissipating pile-anchor structure needs verification. This is done by back-analyzing the test results. The dimensions and material parameters of the back-analysis model are back-calculated from actual test data, following the model setup process detailed in Section 2.2 “Numerical Model Setup”.

**Fig 6:**
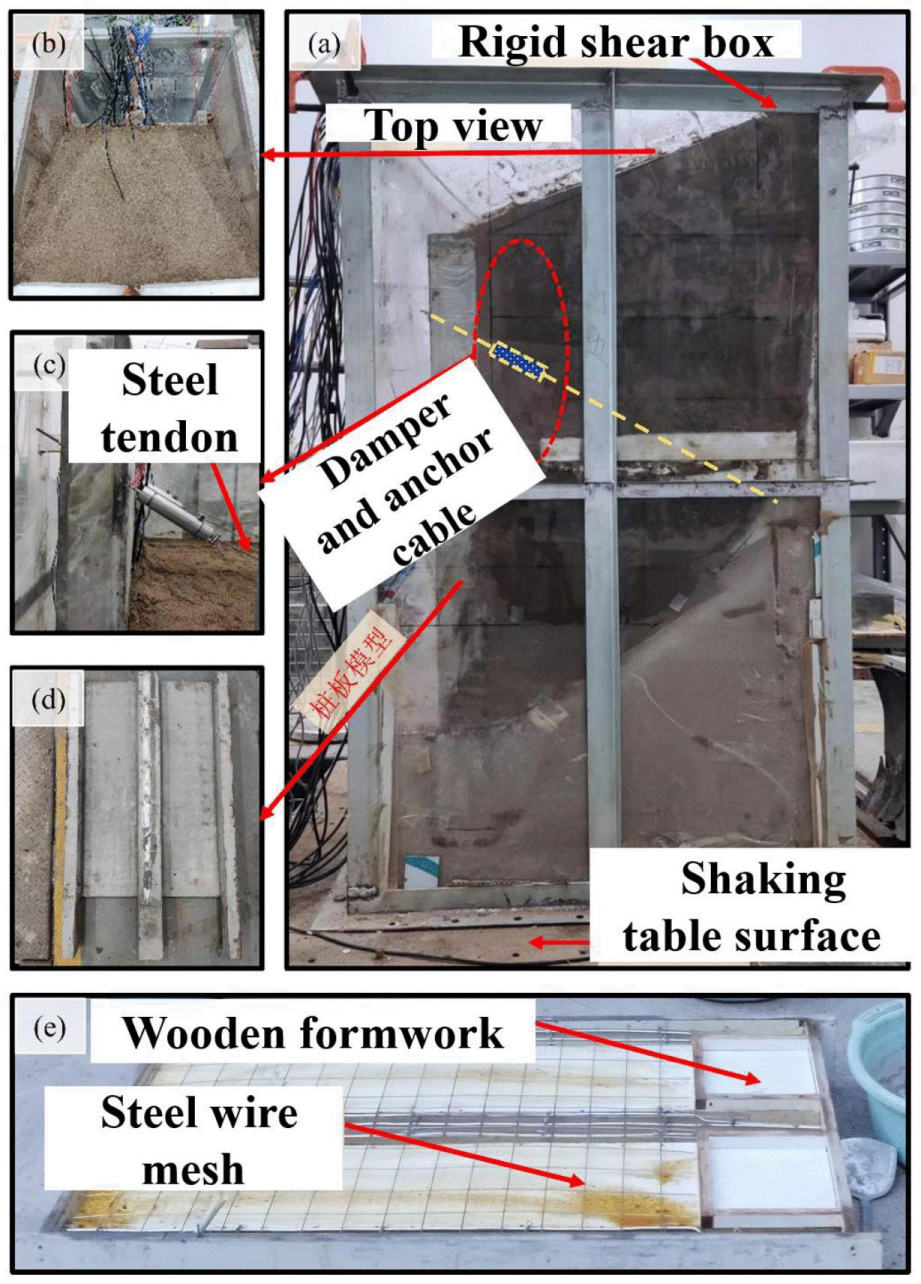
Schematic diagram of test device and test model of energy-dissipating pile-anchor structure.

**Fig 7:**
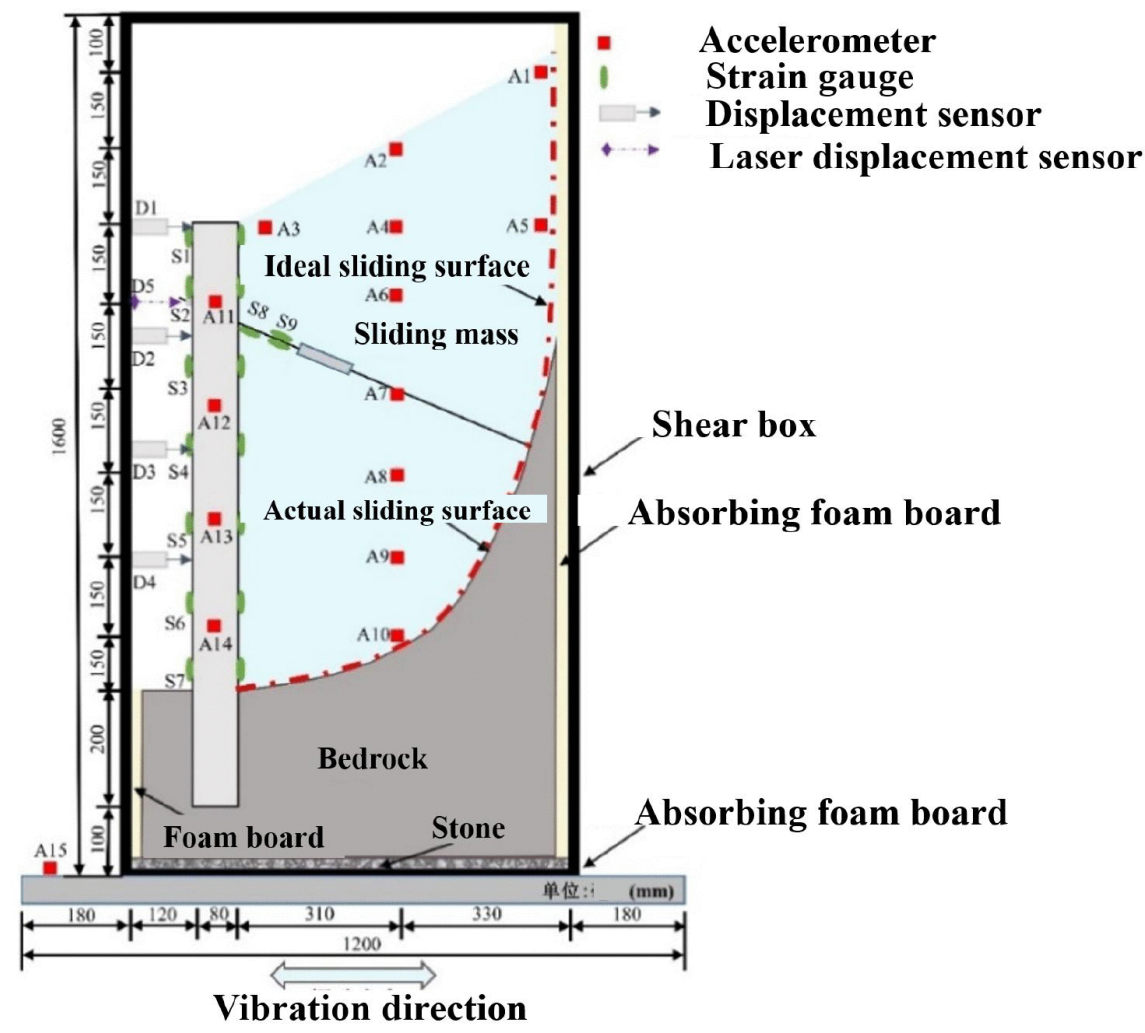
Schematic of the sensor layout for the test model.

To validate the numerical model, this section back-analyzes the energy-dissipating pile-anchor test described [12]. The numerical model uses the prototype dimensions scaled from the 1:10 shaking table test model, with an ideal slip surface replacing the actual test surface. Fig 6 is a schematic diagram of the test device and model of the energy-dissipating pile-anchor structure, while Fig 7 shows the sensor layout of the test model. Further details on the experimental setup and procedures can be found in Liu et al. [12].

Based on material parameters from Liu et al. [12], the bedrock elastic modulus is set to 50 GPa, Poisson’s ratio to 0.2, and density to 2.8 t/m³, consistent with granite parameters. The slide mass uses the Mohr-Coulomb yield criterion with an internal friction angle of 28°, cohesion of 80 kPa, elastic modulus of 40 MPa, corresponding Poisson’s ratio of 0.25, and density of 2.0 t/m³. The tensile strength of the slide mass is calculated as 92 kPa based on $\sigma_{t}\text{=c}\times cot\left(\varphi \right)$ [39]. Concrete uses the CDP model with parameters determined per Section 2.1 “Concrete Damaged Plasticity Model”. The anchor cable elastic modulus is 109 GPa with a Poisson’s ratio of 0.15. The damper damping coefficient is 600 kN/(m/s).

Considering that material plastic damping alone cannot fully suppress non-physical vibrations, this study uses a Rayleigh damping matrix to simulate soil material damping characteristics [40]. Referencing existing research [41,42], the soil damping ratio is set to 0.157. Parameters $\alpha_{R}$ and $\beta_{R}$ for the Rayleigh damping matrix are calculated as:


$$\begin{array}{c} \alpha_{R}=\frac{2\omega_{i}\omega_{j}(\xi_{i}\omega_{j}-\xi_{j}\omega_{i})}{{\omega_{j}}^{\text{2}}-{\omega_{i}}^{\text{2}}} \end{array}$$ (35)

$$\begin{array}{c} \beta_{R}=\frac{2(\xi_{i}\omega_{j}-\xi_{j}\omega_{i})}{{\omega_{j}}^{\text{2}}-{\omega_{i}}^{\text{2}}} \end{array}$$ (36)

Where $\omega_{i}$ and $\omega_{j}$ represent the *i*-th and *j*-th modal frequencies of the model, and $\xi_{i}$ and $\xi_{j}$ are the corresponding damping ratios. This study uses the first two modal frequencies to determine the parameters. Modal analysis gives the first frequency as 7.15 Hz and the second as 8.15 Hz, finally calculating $\alpha_{R}$ and $\beta_{R}$ as 1.19 and 0.02, respectively.

**Fig 8:**
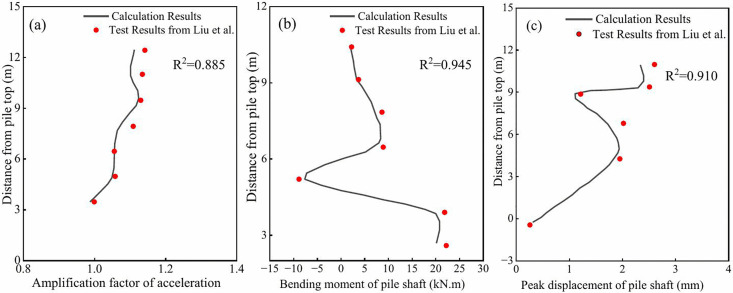
Comparison of numerical model results with test results from Liu et al. [12] (a) Acceleration amplification factor comparison; (b) Pile dynamic bending moment comparison; (c) Pile peak displacement comparison.

It should also be noted that the frequency of the input seismic waves for the back-analysis model was scaled according to the similarity ratio, using a 1.58 Hz sine wave with a peak input acceleration of 0.5g. Fig 8 shows the discrepancy between the calculated results of the back-analysis model and the measured values under the PGA = 0.5g sine wave. The measured values were proportionally converted to prototype responses. The numerical results are slightly smaller, likely because the interaction between the finite and infinite domains and the damping force at the artificial boundary were not considered during ground motion input, reducing the calculated structural seismic response [38]. However, the difference is not significant, and the distribution patterns are largely consistent. This indicates that the numerical model effectively simulates the seismic response characteristics of the energy-dissipating pile-anchor system, demonstrating the model’s validity and the accuracy of the selected constitutive parameters.

## 3. Parametric analysis

### 3.1. Influence of pile-soil relative stiffness

Pile-soil relative stiffness directly affects the force mechanism of anti-slide piles. To study its impact on the seismic performance of energy-dissipating pile-anchor structures, this section analyzes the seismic response characteristics of energy-dissipating anchor cable anti-slide piles with three different stiffnesses, focusing on structural energy dissipation characteristics and pile damage development.

#### 3.1.1. Structural energy dissipation characteristics.

This section uses the built-in ABAQUS output variable EPDDEN (Total energy dissipated per unit volume in the element by plastic deformation) to describe structural energy dissipation. EPDDEN represents the total energy dissipated per unit volume due to plastic deformation, obtained by integrating the area under the material’s stress-strain curve.

**Fig 9:**
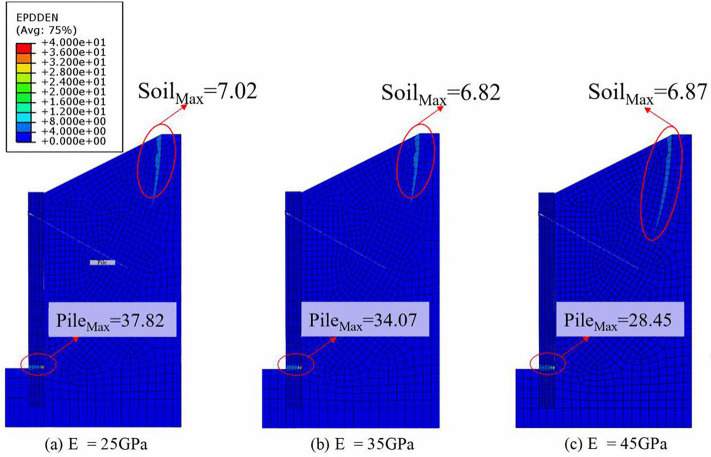
EPDDEN distribution of pile and anchor structures with different stiffness.

Fig 9 shows the EPDDEN distribution contours for energy-dissipating anchor cable anti-slide piles with different stiffnesses under seismic action. Regardless of stiffness, the main energy dissipation points are always at the pile toe and behind the sliding surface. Strengthening these areas can significantly improve structural durability and reliability. As the pile elastic modulus increases, the maximum EPDDEN in the pile gradually decreases, while the maximum EPDDEN in the reinforced slide mass first decreases and then slowly increases. Notably, under the support of the E = 45GPa anti-slide pile, the EPDDEN in the deep soil is slightly larger than that under lower modulus piles. Calculating the total EPDDEN in the slide mass part shows it is largest under the E = 45GPa pile. From an energy perspective, the underlying cause is that excessive pile stiffness diminishes its plastic deformation capacity, leading to inadequate seismic energy absorption. This forces the soil to withstand greater seismic energy, consequently exacerbating soil damage. These findings demonstrate that in pile-anchor structural design, the pile-soil system must be considered as an integrated mechanism. Persistently increasing structural stiffness does not necessarily enhance seismic performance.

#### 3.1.2. Pile damage development.

**Fig 10:**
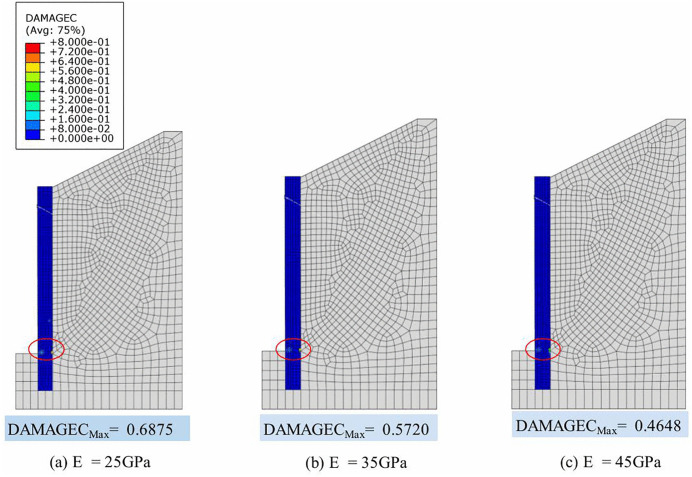
DAMAGEC distribution of pile and anchor structures with different stiffness.

As mentioned in Section 2.1 “Concrete Damaged Plasticity Model”, the concrete damage plasticity model simulates concrete unloading stiffness degradation by introducing damage factors. These factors characterize the degree of concrete damage under compressive or tensile loading, ranging from 0 (no damage) to 1 (severe damage). The compressive damage factor is denoted by the DAMAGEC indicator*d*_*c*_. Fig 10 shows that as the pile-anchor structure stiffness increases, the compressive damage of the anti-slide pile significantly decreases. The maximum damage location remains on the slope-inward side of the pile toe.

**Fig 11:**
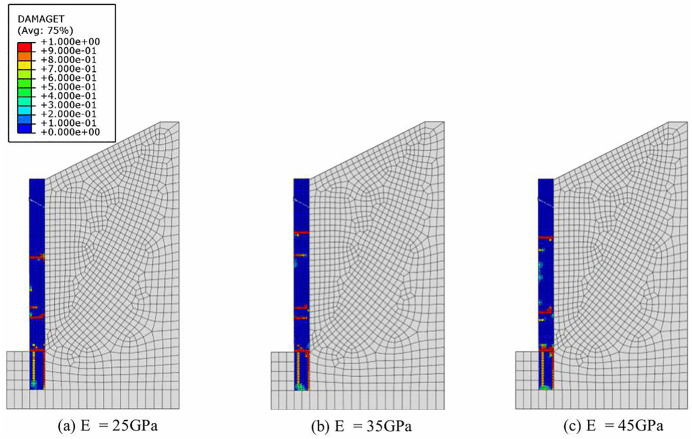
DAMAGET distribution of pile and anchor structures with different stiffness.

As shown in Fig 11, the amplitude of the tensile damage factor (DAMAGET indicator, denoted as *dₜ*) does not exhibit significant reduction with increasing pile stiffness, while the material stiffness degradation (SDEG indicator) is governed by *dₜ*. This demonstrates that merely enhancing material stiffness cannot effectively improve structural seismic resilience, confirming the imperative necessity of incorporating ductile members at critical locations. Furthermore, the observed shift in damage localization with increasing stiffness reflects the influence of pile-soil relative stiffness on structural force distribution.

This paper analyzes pile damage from the perspective of damage energy dissipation. The parameter EDMDDEN (Total energy dissipated per unit volume in the element by damage) represents the total energy dissipated per unit volume due to material damage, typically referring to microscopic failures like crack formation/expansion and void formation. EDMDDEN is obtained by integrating the damage variable over time:


$$\begin{array}{c} EDMDDEN=\underset{O}{\overset{t}{\int }}\sigma :{\dot{\varepsilon }}_{damage}dt \end{array}$$ (37)

Where σ is the stress tensor; ${\dot{\varepsilon }}_{damage}$ is the strain rate tensor due to damage;: represents the double dot product, $A:B=\sum_{i=1}^{n}\sum_{j=1}^{n}A_{ij}B_{ij}$.

**Fig 12:**
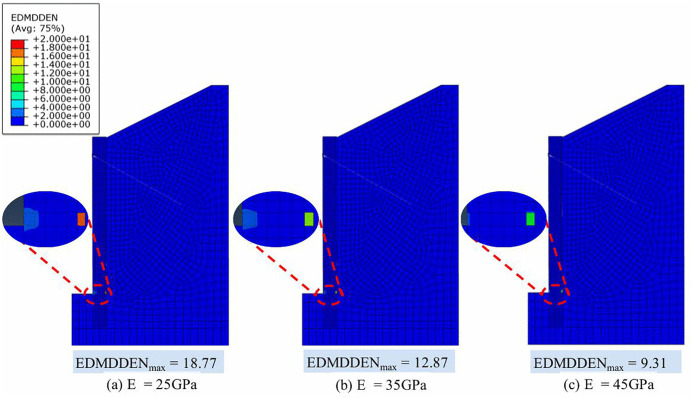
EDMDDEN distribution of pile and anchor structures with different stiffness.

The damage energy dissipation distribution for structures with different stiffnesses is shown in Fig 12. The energy-dissipating pile-anchor structure suffers the most severe damage at the embedded end, consistent with experimental failure phenomena. Increasing pile strength can effectively reduce the energy dissipated by cracking damage, but, as seen before, the rate of component stiffness reduction does not slow down accordingly. This may be because the brittle characteristics of high-strength materials make them more sensitive to cracks, causing slight cracks to quickly affect the overall structural stiffness. This analysis indicates that seismic key nodes require not only high strength but also sufficient ductility to ensure structural safety and stability during earthquakes.

### 3.2. Influence of pile-soil contact roughness

Pile-soil contact roughness significantly affects the seismic performance of energy-dissipating anchor cable anti-slide piles, particularly in lateral friction resistance, pile force state, and failure mode. Understanding its mechanism is crucial for structural optimization. To deeply explore its impact on energy dissipation capacity and failure characteristics, this study constructed four numerical models with different pile-soil contact friction coefficients.

#### 3.2.1. Pile damage development.

**Fig 13:**
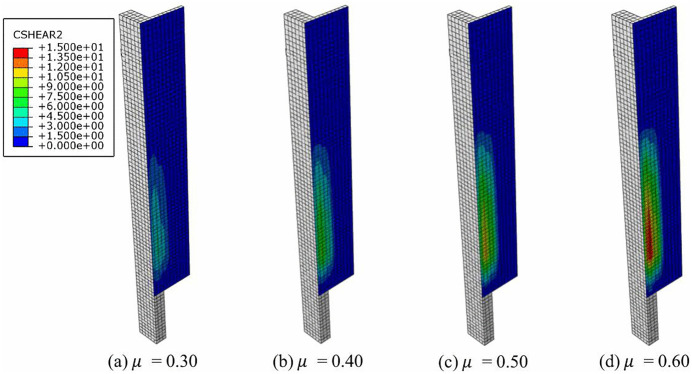
Friction coefficient of energy-dissipating pile anchor structures with different pile-soil contact friction coefficients.

Fig 13 shows the variation of pile-back friction force with different pile-soil contact roughness. As the friction coefficient increases, the lateral friction resistance on the pile back grows significantly. This change alters the eccentric axial force on the pile, consequently affecting the pile’s failure mode.

**Fig 14:**
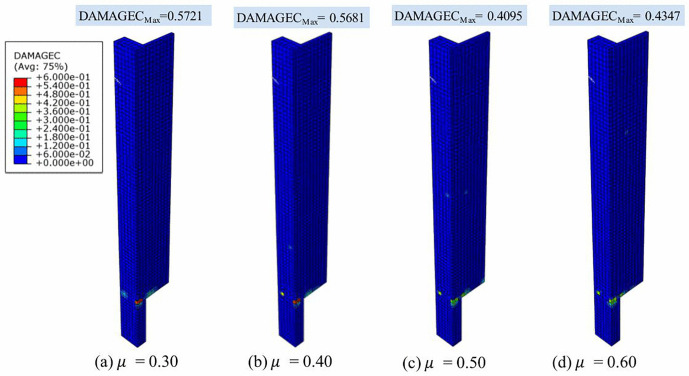
Compressive damage distribution of energy-dissipating pile anchor structures with different pile-soil contact friction coefficients.

Fig 14 shows the compressive damage distribution for different friction coefficients. The maximum compressive damage first decreases and then increases with increasing friction coefficient. This phenomenon is mainly due to changes in the eccentric axial force and corresponding additional bending moment caused by lateral friction. These changes alter the bending moment distribution at the pile toe section during vibration, gradually reducing damage in the inward compression zone while increasing compressive strain on the free-face side. Therefore, when the friction coefficient *μ* is 0.6, the maximum compressive damage increases slightly.

**Fig 15:**
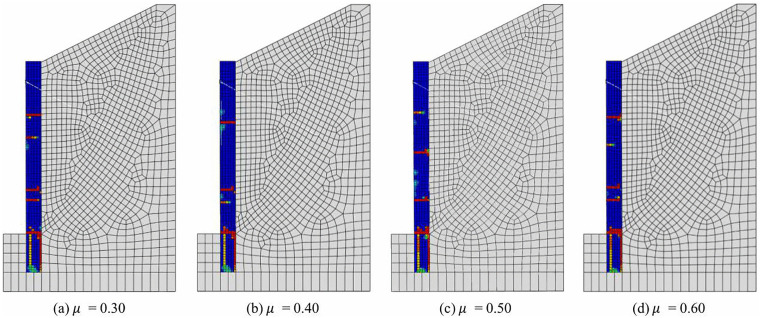
Tensile damage distribution of energy-dissipating pile anchor structures with different pile-soil contact friction coefficients.

**Fig 16:**
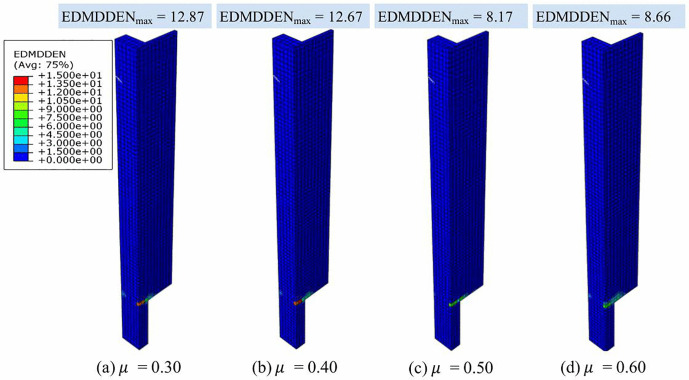
EDMDDEN distribution of energy-dissipating pile anchor structures with different pile-soil contact friction coefficients.

As shown in Fig 15, changes in the additional bending moment from lateral friction also noticeably affect pile tensile damage. On one hand, the damage distribution location changes due to altered bending moment distribution. On the other hand, combined with the pile damage energy distribution (Fig 16), the overall pile tensile damage first decreases and then increases with changing friction coefficient. This suggests that adjusting the pile-soil interface roughness can achieve a dynamic balance between friction resistance and seismic force, reducing structural damage during earthquakes.

#### 3.2.2. Structural energy dissipation characteristics.

**Fig 17:**
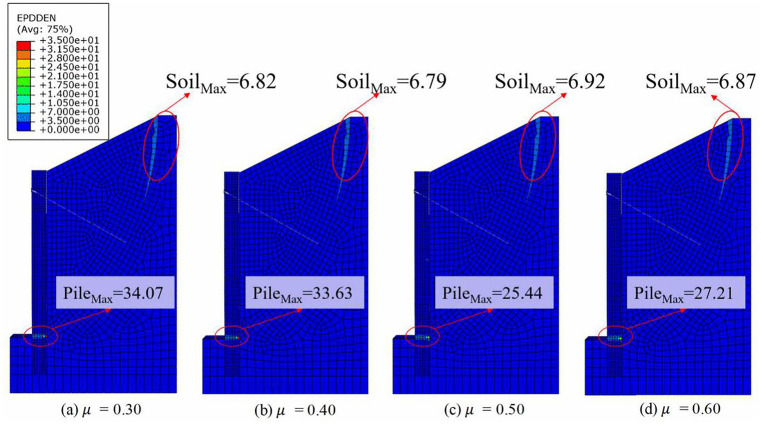
EPDDEN distribution diagram of pile anchor structures with different pile-soil contact friction coefficients.

Fig 17 presents the EPDDEN distribution of energy-dissipating pile-anchor structures with varying pile-soil interface friction coefficients. As observed, the plastic dissipated energy in the pile initially decreases and subsequently increases with rising friction coefficient, while the plastic dissipated energy in the soil remains relatively unchanged. This phenomenon can be interpreted from an energy perspective: the enhanced pile-soil frictional interaction dissipates a portion of seismic energy through friction, thereby mitigating plastic damage in the pile. This observation aligns consistently with the established patterns of damage evolution.

### 3.3. Influence of structural member ductility

Previous test analysis showed that using ECC ductile members can significantly improve structural deformation capacity and seismic performance [12]. To further explore the impact of ECC members on the energy dissipation capacity and damage characteristics of energy-dissipating pile-anchor structures, this study established numerical models of ductile pile-anchor structures (with ECC members) under different seismic intensities and compared them with energy-dissipating pile-anchor structures using ordinary concrete (RC) members under the same intensities.

#### 3.3.1. Pile damage development.

**Fig 18:**
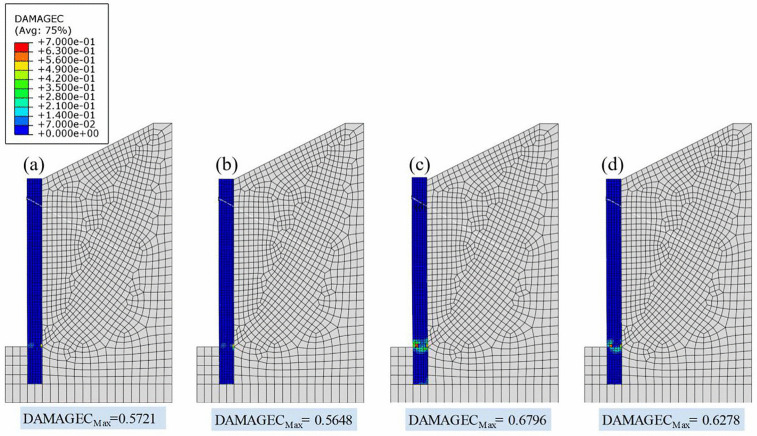
Compressive damage distribution clouds of ductile pile anchor structures and energy-dissipating pile anchor structures under different magnitudes (a) RC member PGA = 0.5g; (b) ECC member PGA = 0.5g; (c) RC member PGA = 0.7g; (d) ECC member PGA = 0.7g.

**Fig 19:**
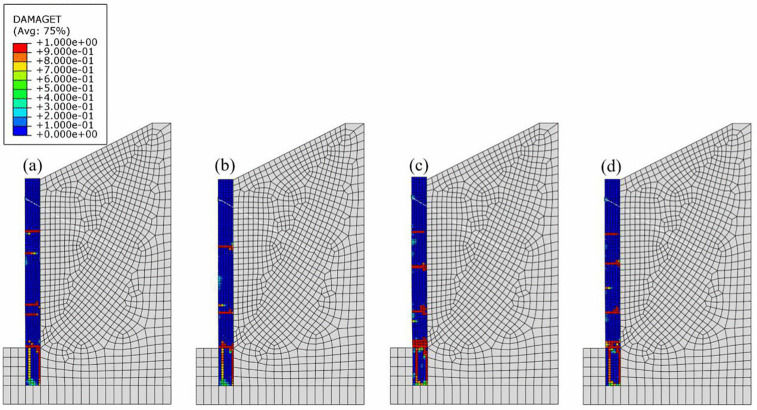
Tensile damage distribution of ductile pile-anchor structure and energy-dissipating pile-anchor structure under different seismic magnitudes (a) RC member PGA = 0.5g; (b) ECC member PGA = 0.5g; (c) RC member PGA = 0.7g; (d) ECC member PGA = 0.7g.

**Fig 20:**
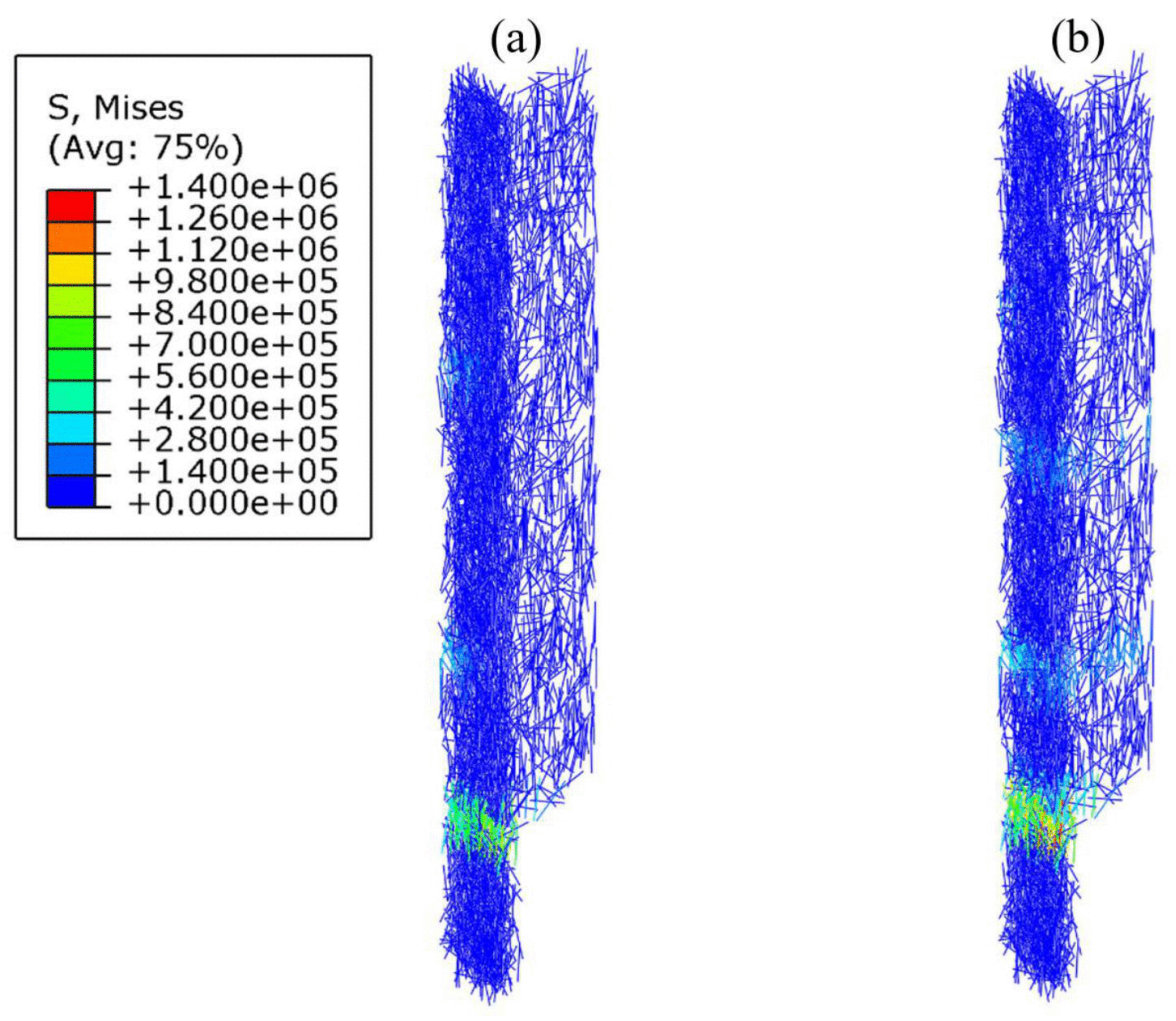
Distribution of internal fiber stress in ductile pile-anchored structures under different earthquake magnitudes (a) Fiber-reinforced member PGA = 0.5g; (b) Fiber-reinforced member PGA = 0.7.

Fig 18 shows that using ECC members reduces component compressive damage, and this effect enhances noticeably with increasing seismic intensity. Fig 19 further demonstrates the advantage of ECC in limiting damage development under different intensities. Compared to traditional RC members, ECC members have a wider distribution of damage but lower local damage severity. This is primarily due to the bridging effect of fibers within the ECC, which transfer stress across cracks (Fig 20), promoting more uniform stress distribution and avoiding excessive damage at initial crack locations, thereby enhancing the overall performance of ECC members.

**Fig 21:**
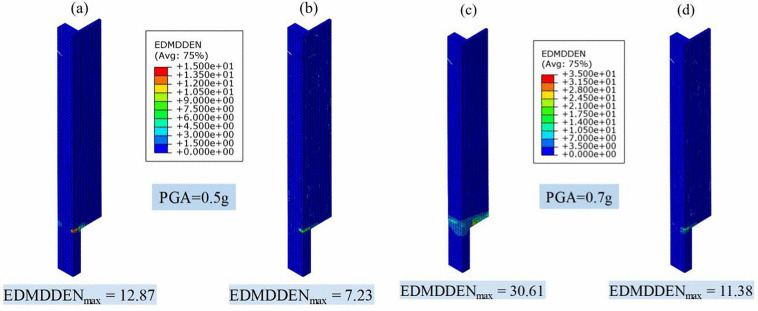
EDMDDEN distribution cloud map of ductile pile-anchor structure and energy-dissipated pile-anchor structure under different seismic magnitudes (a) RC member PGA = 0.5g; (b) ECC member PGA = 0.5g; (c) RC member PGA = 0.7g; (d) ECC member PGA = 0.7g.

Fig 21 describes the damage dissipation energy distribution for ductile and energy-dissipating structures under different intensities. Clearly, the damage level in ECC members is much lower than in RC members, and the gap widens with increasing intensity. Calculating the total damage dissipation energy (see Tables 6 and 7) reveals that under PGA = 0.5g, the damage dissipation energy of ECC members is only 77.6% of RC members, and this ratio decreases to 33.8% under PGA = 0.7g. These phenomena fully demonstrate the excellent damage control capability and seismic performance of ECC members, making them particularly suitable for slope reinforcement under strong earthquakes.

**Table 6:** Total plastic dissipation energy and damage dissipation sum of ductile pile-anchor structure and energy-dissipating pile-anchor structure under earthquake action of PGA = 0.5.

Physical Quantity	Energy-Dissipating Pile-Anchor Structure	Ductile Pile-Anchor Structure
Total Pile Plastic Dissipation Energy (EPDDEN)	1.448kJ	1.045 kJ
Total Pile Damage Dissipation Energy (EDMDDEN)	0.250 kJ	0.194 kJ
Damper Energy Dissipation	10.16 kJ	8.307 kJ

**Table 7:** Total plastic dissipation energy and damage dissipation sum of ductile pile-anchor structure and energy-dissipating pile-anchor structure under earthquake action of PGA = 0.7.

Physical Quantity	Energy-Dissipating Pile-Anchor Structure	Ductile Pile-Anchor Structure
Total Pile Plastic Dissipation Energy (EPDDEN)	4.314 kJ	2.200 kJ
Total Pile Damage Dissipation Energy (EDMDDEN)	1.050 kJ	0.355 kJ
Damper Energy Dissipation	6.156 kJ	17.597 kJ

#### 3.3.2. Structural energy dissipation characteristics.

**Fig 22:**
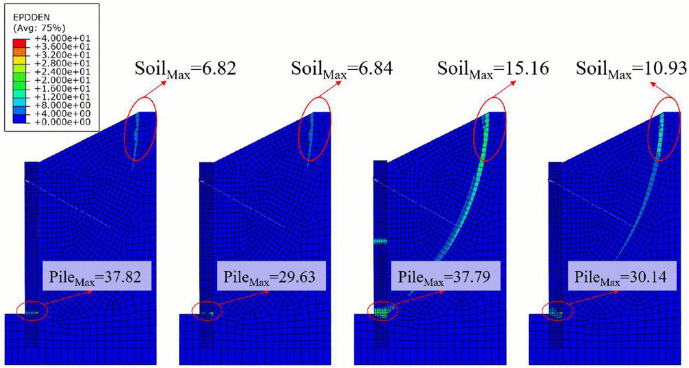
EPDDEN distribution of ductile pile-anchor structure and energy-dissipated pile-anchor structure under different seismic magnitudes (a) RC member PGA = 0.5g; (b) ECC member PGA = 0.5g; (c) RC member PGA = 0.7g; (d) ECC member PGA = 0.7g.

Fig 22 shows the differential distribution of plastic dissipated energy between ductile pile-anchor structures and energy-dissipating pile-anchor structures under varying seismic intensities. It can be observed that at lower intensity levels, the incorporation of ECC members significantly reduces the maximum plastic dissipated energy in the pile, while exhibiting negligible impact on the maximum plastic dissipated energy in the sliding mass. With increasing seismic intensity, the maximum plastic strain in RC members shows limited growth; however, substantial plastic strains develop at multiple locations along the pile, resulting in an increase in total plastic dissipated energy. Concurrently, the maximum plastic dissipated energy in the sliding mass supported by RC members rises sharply, with significant plastic strains distributed across the entire slip surface, leading to an elevated total plastic dissipated energy in the sliding mass. This indicates that RC members experience severe damage under strong seismic motions, consequently failing to effectively reinforce the sliding mass and permitting sliding along the predetermined slip surface. In contrast, ECC members not only mitigate plastic development in the pile under strong earthquakes but also substantially suppress the growth of plastic dissipated energy in the sliding mass. The energy dissipation of damping devices during vibration, obtained by calculating the hysteretic loop area from damper force-displacement curves (as presented in Tables 6 and 7), demonstrates that the ductile characteristics of ECC members maintain structural integrity under strong seismic conditions, enabling full activation of damping devices and thereby significantly enhancing both the energy dissipation capacity and seismic performance of the support system.

## 4. Conclusions

This paper deeply analyzed the energy dissipation and damage evolution laws of energy-dissipating and ductile pile-anchor structures under seismic action using numerical methods. The study employed the CDP model to simulate concrete stress-strain response, combined with a 3D microscopic model to accurately characterize the ductile characteristics of fiber-reinforced concrete (ECC), and used a static-dynamic artificial boundary conversion method to improve dynamic analysis accuracy. By establishing a series of numerical models, the study systematically analyzed the effects of pile stiffness, pile-soil contact roughness, and ECC ductile members on the damage development and energy dissipation characteristics of energy-dissipating pile-anchor structures. The main conclusions are as follows:

(1) The numerical model, incorporating the CDP constitutive model with parameters derived from material tests and calibrated against shaking table test results accurately captures the dynamic response characteristics of the energy-dissipating pile-anchor system. The combination of the Concrete Damaged Plasticity model and a 3D fiber-reinforced concrete model effectively simulates the damage behavior of both conventional and Engineered Cementitious Composite (ECC) members.
(2) Pile stiffness plays a critical role in load sharing between the pile and the soil. While increasing pile stiffness reduces pile plastic damage, excessive stiffness transfers more seismic energy to the surrounding soil, exacerbating soil failure. This highlights the necessity of balancing strength and deformation capacity in seismic design.
(3) Pile-soil interface roughness significantly influences the pile’s failure mode and energy dissipation. An optimal friction coefficient can enhance energy dissipation through interface friction, thereby reducing structural damage. This finding provides a basis for optimizing pile-soil interaction in design.
(4) ECC members exhibit superior seismic performance compared to ordinary concrete. They substantially reduce structural damage (damage dissipation energy reduced by up to 52.4% under strong shaking) and maintain integrity under large deformations, enabling full activation of damping devices and improving overall system safety. This demonstrates the great potential of ECC for enhancing the resilience of slope support structures in earthquake-prone regions.

These findings advance the understanding of energy dissipation in pile-anchor systems and offer practical guidance for their seismic optimization, contributing to more resilient geotechnical infrastructure. However, this numerical study used an infinite element as the artificial truncation boundary. Although static-dynamic boundary conversion improved accuracy, some discrepancies with experimental results remain because interactions between finite and infinite domains and the damping force of the artificial boundary were not considered during ground motion input.

## Supporting information

S1 File Data. (XLS)
